# Midazolam Exerts Sedative Effects by Differentially Modulating Cortical Pyramidal Neurons and PV Interneurons

**DOI:** 10.1002/cns.71049

**Published:** 2026-07-24

**Authors:** Yao Sun, Dijia Wang, Kaibin Wu, Mengyu Yin, Peiwen Tang, Fengxian Li, Junlei Chang, Feixue Liang, Hong‐Fei Zhang

**Affiliations:** ^1^ Department of Anaesthesiology Zhujiang Hospital, Southern Medical University Guangzhou China; ^2^ Institute of Perioperative Medicine and Organ Protection, Zhujiang Hospital, Southern Medical University Guangzhou China; ^3^ Key Laboratory of Mental Health of the Ministry of Education, Guangdong‐Hong Kong‐Macao Greater Bay Area Center for Brain Science and Brain‐Inspired Intelligence, Guangdong‐Hong Kong Joint Laboratory for Psychiatric Disorders, Guangdong Province Key Laboratory of Psychiatric Disorders, Guangdong Basic Research Center of Excellence for Integrated Traditional and Western Medicine for Qingzhi Diseases, School of Biomedical Engineering, Southern Medical University Guangzhou China; ^4^ Guangdong Provincial Key Laboratory of Shock and Microcirculation Southern Medical University Guangzhou China; ^5^ State Key Laboratory of Biomedical Imaging Science and System Institute of Biomedicine and Biotechnology, Shenzhen Institute of Advanced Technology, Chinese Academy of Sciences Shenzhen Guangdong China

**Keywords:** midazolam, parvalbumin‐positive interneurons, pyramidal neurons, sedation, γ‐Aminobutyric acid type a receptors

## Abstract

**Aims:**

Midazolam is a sedative that acts on γ‐aminobutyric acid type A receptors, but whether its effects are region‐specific, especially in the neocortex, and the mechanisms underlying these effects remain unclear.

**Methods:**

Using microendoscopic Ca^2+^ imaging and whole‐cell recording in brain slices, we systematically investigated the effects of midazolam on excitatory pyramidal neurons and inhibitory parvalbumin‐positive interneurons (PV^+^) in the mouse auditory cortex (AC) and anterior cingulate cortex (ACC).

**Results:**

Midazolam dose‐dependently suppressed pyramidal neurons more potently in the AC than in the ACC (Evoked: pyramidal neurons: 0.65 ± 0.30 vs. 1.36 ± 0.34, *p* < 0.0001; Spontaneous: pyramidal neurons: 1.49 ± 0.48 vs. 2.25 ± 0.42, *p* = 0.0006). In slices, midazolam reduced the action potential firing of pyramidal neurons in a concentration‐dependent manner, with greater sensitivity in the AC. In contrast, PV^+^ neurons showed little change in intrinsic excitability in either region. Functional assays suggested that midazolam responsiveness was associated with GABRA1 expression, which was higher in AC pyramidal neurons than in those of the ACC (1.77 ± 0.55 vs. 1.01 ± 0.36, *p* = 0.002), while PV^+^ neurons showed no significant regional difference in GABRA1 expression.

**Conclusion:**

Midazolam suppresses cortical excitatory neurons in a region‐ and cell‐type‐dependent manner that is associated with differential expression of GABRA1.

## Graphical abstract

We show that midazolam produces region‐ and cell‐type‐dependent effects, with stronger suppression in the auditory cortex than in the anterior cingulate cortex and greater sensitivity in pyramidal neurons than in PV+ interneurons. Differential GABRA1 expression is associated with this regional variation in midazolam sensitivity. These findings indicate that cortical responses to midazolam are not uniform across regions or distinct neuronal populations.

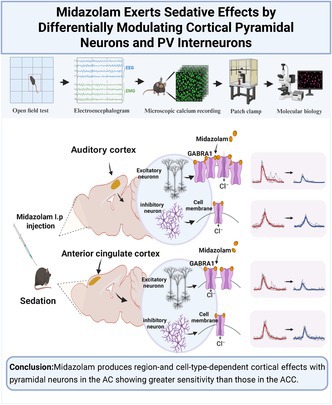

## 1 Introduction

Midazolam, a widely used benzodiazepine sedative, acts primarily by enhancing inhibitory neurotransmission via γ‐aminobutyric acid type A receptors (GABAARs) [1, 2]. Due to its rapid onset and favorable pharmacokinetics, it is commonly employed for preoperative sedation, anesthesia induction, and the treatment of acute agitation and seizures [3, 4]. Despite its widespread use, the neural circuit mechanisms underlying midazolam's sedative effects remain incompletely understood.

In recent years, clinical electroencephalography and functional magnetic resonance imaging studies have contributed to the formation of a “hierarchical processing” model of anesthetic/sedative action [5]. This model posits that during altered states of consciousness induced by different drugs, functional connectivity in higher‐order associative cortices is often disrupted, while activity varies across functional areas such as primary sensory cortices and cognition‐related nuclei, reflecting differential sensitivity of brain regions to anesthetic agents [6, 7, 8, 9, 10]. In contrast to general anesthetics, clinical sedatives allow patients to remain in a state in which they can be aroused by external stimuli. This observation led us to hypothesize that sedatives may exert differential inhibitory effects across brain regions. The primary auditory cortex (AC) serves as a key area for processing auditory information and regulating arousal driven by auditory inputs [11]. Studies have shown that anesthetics significantly reduce neuronal activity in the AC [12]. The anterior cingulate cortex (ACC), as an integrative frontal lobe nucleus, plays important roles in consciousness, cognitive processes, and motivation and has been shown to be involved in the central mechanisms of various anesthetic drugs [13, 14]. Whether midazolam exhibits region‐specific effects and differentially influences distinct neuronal types—such as excitatory pyramidal neurons and inhibitory interneurons—remains incompletely understood.

Pyramidal neurons form the primary excitatory output of the neocortex and are key targets in the sedative modulation of cortical function [15, 16, 17]. In contrast, parvalbumin‐positive (PV^+^) interneurons mediate fast‐spiking inhibition, regulating the timing and synchrony of cortical networks [12, 18]. The balance between excitatory and inhibitory (E/I) activity is essential for normal cortical function and represents a key target for sedative action [19, 20]. Disruption of this E/I balance contributes to altered states of consciousness during sedation and anesthesia.

At the molecular level, GABAARs are heteropentameric chloride channels formed by specific subunits, including α1 (GABRA1), α2 (GABRA2), and α3 (GABRA3), which determine receptor pharmacodynamics and regional distribution [21]. Midazolam binds to the α/γ subunit interface. Growing evidence indicates that differential expression of GABAARs may contribute to region‐ and cell‐type‐specific responses to benzodiazepines [22]. However, direct experimental evidence linking GABAAR distribution to midazolam's effects within specific cortical circuits remains limited.

In this study, we systematically investigated the region‐ and cell‐type‐specific effects of midazolam on cortical excitatory and inhibitory neurons, with a focus on the primary auditory cortex (AC) and anterior cingulate cortex (ACC). Microendoscopic Ca^2+^ imaging (miniscope) was used to quantify sound‐evoked and spontaneous activity in pyramidal neurons and PV^+^ interneurons of awake mice. Acute slice patch‐clamp recordings were used to assess intrinsic neuronal properties during midazolam application. Finally, molecular profiling and functional assessment of GABAAR expression were performed, with a focus on GABRA1. We show that midazolam preferentially suppresses pyramidal neuron activity in a region‐ and cell‐type‐specific manner and that this pattern may be associated with regional differences in GABRA1 expression. This provides insights into the cortical effects of midazolam.

## 2 Materials and Methods

### 2.1 Animal Preparation

Animals were housed in a temperature‐ and humidity‐controlled vivarium with ad libitum access to food and water and a 12‐h light/dark cycle (lights on at 08:00). Male and female wild‐type C57BL/6J mice (Southern Medical University Animal Centre, Guangzhou, China) and transgenic PV‐Cre, PV‐Cre; Ai14, Thy1‐Cre, and Thy1‐Cre; Ai14 mice (The Jackson Laboratory) were used.

### 2.2 Drug

Midazolam injection: Jiangsu Enhua Pharmaceutical Co. Ltd., specification: 1 mL/5 mg, batch number: MD200901.

### 2.3 Microendoscope Ca^2+^ Imaging Recordings

The surgical procedures were performed as described in previous studies [23, 24, 25]. To image neurons in the AC or ACC, AAV2/1‐hsyn‐FLEX‐GCaMP8s‐WPRE (100–200 nL) was injected unilaterally into AC (AP: −2.5 to −3.2 mm; ML: +4.05 mm; DV: −2.4 mm) or ACC (AP: +1.4 to +1.9 mm; ML: 0.35 mm; DV: −2.0 mm) of Thy1‐Cre mice for excitatory neurons recordings or PV‐Cre mice for PV^+^ interneuron recordings. Two weeks after virus injection, a gradient refractive index (GRIN) lens (0.5‐ or 1.0‐mm diameter and 4.2 mm length; Inscopix) was implanted above the injection site and secured to the skull using super glue and dental cement. Mice received a subcutaneous injection of ketoprofen (4 mg·kg^−1^) during surgery. Postoperative care included administration of ibuprofen in drinking water (30 mg·kg^−1^) for 4 days. After a recovery period of 1–4 weeks, the microendoscope, together with a titanium baseplate, was placed above the lens. The microendoscope position was adjusted until cells and blood vessels appeared sharp in the focal plane. The baseplate was then secured in this position with dental cement. All mice were handled and habituated for more than 3 days before imaging experiments.

Before imaging began, the mice were temporarily head‐anchored and fitted with a miniscope to examine their response to auditory stimuli. A 15‐ to 30‐min acclimation period was implemented. Sound stimulation (70 dB white noise burst) commenced 2.5 s after the initiation of recording and lasted for 2 s, while calcium signals were continuously acquired for a total of 10 s. Calcium signals were recorded at a frequency of 25 Hz. All imaging parameters were kept consistent across trials for each mouse. Image stacks were saved in RAW format. Frames were spatially down‐sampled and high‐pass filtered (40 μm stopband). Session frames were concatenated and motion‐corrected using NoRMCorre. Constrained non‐negative matrix factorization (CNMF‐E) was applied to extract neuronal ROIs and raw fluorescence traces (F); non‐neuronal components (identified by non‐somatic morphology) were manually excluded. Δ*F/F* was calculated and z‐scored for each neuron.

### 2.4 Preparation of Acute Brain Slices

Detailed experimental procedures have been described previously [26]. C57BL/6J mice (6–8 weeks old) were anesthetized with isoflurane. Following decapitation, the brains were rapidly extracted and immersed in ice‐cold oxygenated dissection solution (in mM: 60 NaCl, 3 KCl, 7 MgCl_2_, 0.5 CaCl_2_, 25 NaHCO₃, 1.25 NaH_2_PO₄, 10 glucose and 115 sucrose). Coronal slices (300 μm thick) containing the AC and ACC were cut using a vibratome (Leica VT1000S, Germany) in the same solution. Slices were incubated for more than 30 min at 34°C in oxygenated artificial cerebrospinal fluid (aCSF) (in mM: 126 NaCl, 2.5 KCl, 1 MgCl_2_, 2 CaCl_2_, 26 NaHCO₃, 1.25 NaH_2_PO₄, 10 glucose, 0.5 ascorbic acid, and 2 sodium pyruvate). After incubation, the slices were transferred to a recording chamber maintained at 25°C ± 1°C for electrophysiological recordings. All solutions were continuously oxygenated with 95% O_2_/5% CO_2_ (pH 7.4, 300–320 mOsm·L^−1^).

### 2.5 Electrophysiological Recording

Recordings were performed using an upright fluorescence microscope (Olympus BX51WI) equipped with an infrared light source. Glass pipettes (7–10 MΩ resistance) were prepared using a micropipette puller (P‐2000, Sutter Instrument, USA). For current‐clamp recordings, pipettes were filled with potassium‐based internal solution (in mM): 125 K‐gluconate, 10 HEPES, 10 EGTA, 4 Mg‐ATP, 0.3 GTP, 2 KCl, 0.1 CaCl_2_, and 8 Na‐phosphocreatine, pH 7.2 (adjusted with KOH). Action potentials (APs) were evoked by current injections (−150 to + 350 pA, in 50 pA increments, 1 s duration) in current‐clamp mode to assess neuronal excitability. During the recording, baseline AP levels were recorded for at least 3 min before the drugs were perfused. Midazolam was then bath‐applied for 10 min, followed by repeated measurements. One neuron per slice was recorded because of the prolonged washout kinetics of the drug. The first AP at rheobase (defined as the minimal current of infinite duration, experimentally limited to 300 ms, required to generate an AP) was analyzed for AP half‐width and AP onset. Series resistance was compensated online (50%–70%). Data were excluded if the series resistance drift exceeded 15% or absolute resistance exceeded 20 MΩ. Signals were acquired using a MultiClamp 700B amplifier and Digidata 1440A digitizer (Axon Instruments, USA). Data were analyzed with pClamp 10.7 (Molecular Devices, USA) and MiniAnalysis 6.0 (Synaptosoft, USA).

### 2.6 Open Field Test

The open field test was performed in an uncovered cubic arena (40 × 40 × 40 cm), as previously described [27]. Mice were allowed to freely explore the arena for 5 min. During formal testing, baseline locomotion was first recorded for 30 min. Following administration of different doses of midazolam, behavior was recorded for an additional 60 min. A digital camera mounted directly above the arena captured all sessions. Locomotor parameters (speed, travel distance, and movement duration) and trajectory plots were analyzed using SMART V3.0 video tracking software (Planlab) for all behavioral trials.

### 2.7 Electroencephalogram Recording and Analysis

Electroencephalograms (EEGs) were recorded in mice as previously described [12]. Briefly, electrodes were connected to a 32‐channel head‐stage via a five‐wire connector and controlled by an Open Ephys acquisition board. Raw EEG data were sampled at 1000 Hz and band‐pass filtered (0.5–48 Hz). Power spectral density (PSD) of the entire segment was computed using Welch's method. The power within each frequency band was then normalized to the total power across the 0–48 Hz range. Frequency bands were defined as delta (0.5–4 Hz), theta (4–8 Hz), alpha (8–15 Hz), beta (15–25 Hz), and gamma (25–48 Hz).

### 2.8 Real‐Time qPCR (RT‐qPCR)

Total RNA was extracted using TRIzol reagent (Vazyme Biotech, Nanjing, China) according to the manufacturer's instructions, as previously described [28]. RNA purity and concentration were measured using a NanoDrop 2000 spectrophotometry (Thermo Fisher Scientific, USA), with A260/A280 ratios of 1.8–2.0 considered acceptable. cDNA was synthesized using HiScript III RT SuperMix (Vazyme Biotech, Nanjing, China). RT‐qPCR was performed using ChamQ Universal SYBR Green qPCR Master Mix (Vazyme Biotech, Nanjing, China) on a LightCycler 96 System (Roche Diagnostics, Basel, Switzerland). For mouse genes, the following primer sequences were used:

β‐Actin forward primer: GAAGTGTGACGTTGACATCCG, β‐Actin reverse primer: GTCAGCAATGCCTGGGTACAT.

GABRA1 forward primer: TGCCAGAAATTCCCTCCCGAAG, GABRA1 reverse primer: CCATCCCACGCATACCCTCTC.

GABRA2 forward primer: AGAGAATCGGTGCCAGCAAGAAC, GABRA2 reverse primer: AAGCCACTTTCGGGAGGGAATTTC.

GABRA3 forward primer: TGGACGGTTCATAGCCGTCTG, GABRA3 reverse primer: TGCCTTCCCAAGCCCAACTC.

### 2.9 Cell Culture and Cell Transfection

Detailed experimental procedures have been described previously [29]. HEK293T cells were obtained from the American Type Culture Collection (ATCC, USA) and cultured in Dulbecco's Modified Eagle Medium (DMEM) supplemented with 10% fetal bovine serum (FBS) and 1% penicillin/streptomycin (P/S). For GABRA1/2 overexpression, HEK293T cells were transfected with GABRA1/2 plasmids or an empty vector (pcDNA3.1; MiaoLing Plasmid Platform, China) using JetOPTIMUS Transfection Reagent (Polyplus, Cat. #0000003010) when cells reached 50%–60% confluence. Plasmids were diluted in DMEM without FBS or P/S. Equal volumes of diluted transfection reagent and plasmid solution were mixed and incubated at room temperature for 15 min before being added to the cells. Cells were used for subsequent experiments 24–48 h after transfection.

### 2.10 Calcium Signal Detection In Vitro

Transfected cells were loaded with the intracellular calcium indicator Calbryte 520 AM (AAT Bioquest, USA; Cat. #20650) [30] in 1× Hank's Balanced Salt Solution (HBSS; containing CaCl_2_, MgCl_2_, and no phenol red; Gibco, Waltham, MA, USA; Cat. #14025092) for 30 min at 37°C. Samples were washed three times with HBSS before imaging was performed continuously for 30 min using a fluorescence microscope (Zeiss Axio Imager Z2 with ApoTome.2, Germany).

### 2.11 Immunofluorescence Staining

Detailed experimental procedures have been described previously [31]. Mice were perfused with ice‐cold PBS. Brains were collected, fixed in 4% paraformaldehyde (PFA) dissolved in phosphate‐buffered saline (PBS, pH 7.2) for 2 h, and processed for dehydration. Samples were dehydrated through a graded ethanol series and embedded in paraffin. Coronal sections (10‐μm‐thick) were cut using a Leica CM 1950 microtome (Leica Biosystems, Germany), heated at 60°C for 40–60 min, dewaxed in xylene, and rehydrated through graded ethanol solutions. Antigen retrieval was performed with sodium citrate solution (Solarbio, China, Cat. #C1032). After three 10‐min washes with PBS, sections were blocked with 10% goat serum at room temperature for 1 h, then incubated overnight at 4°C with primary antibodies: anti‐GABRA1 (1:200, proteintech, China, cat. #12410–1‐AP). On the following day, samples were incubated with fluorophore‐conjugated secondary antibodies (1:1000; Jackson ImmunoResearch, USA) for 1 h at room temperature. Sections were mounted using DAPI‐containing antifade medium (Solarbio, Cat. #S2110) and imaged with a Zeiss Axio Imager Z2 fluorescence microscope equipped with ApoTome.2 (Carl Zeiss, Germany). During whole‐cell recordings, neurons were intracellularly infused with a solution containing 0.3% biocytin (Sigma‐Aldrich, Cat. #B4261). Following 20 min of voltage‐clamp recording, slices were fixed in 4% formaldehyde at 4°C until processing. Slices were washed three times with PBS (10 min per wash) and blocked with 0.3% Triton X‐100 in PBS for 3 h. After additional PBS washes (three times, 10 min each), slices were incubated with streptavidin‐Cy3 conjugate (1:200; Molecular Probes, USA; Cat. #434315) at 4°C overnight.

### 2.12 Statistical Analysis

Concentration‐response curves for midazolam‐mediated suppression of firing frequency in AC and ACC pyramidal neurons were fitted using a four‐parameter logistic model through nonlinear regression. For patch‐clamp data, differences between control and midazolam groups were assessed using two‐way repeated‐measures ANOVA with Bonferroni post hoc test, unpaired *t*‐test, or Mann–Whitney *U* test. Comparisons among ≥ 3 groups used one‐way ANOVA followed by Tukey post hoc analysis. Normality was assessed using the Shapiro–Wilk test. Data are expressed as mean ± SD or EC50 (95% CI) unless otherwise noted. All statistical tests were two‐tailed, with *p* < 0.05 considered statistically significant. Data accuracy was verified against original recordings to prevent overinterpretation. Analyses used GraphPad Prism 8.0 (GraphPad Software) and SPSS 22.0 (IBM Corp). Detailed methods can be found in the Supporting Information.

## 3 Results

### 3.1 Midazolam Produces Dose‐ and Time‐Dependent Sedation In Vivo

**FIGURE 1:**
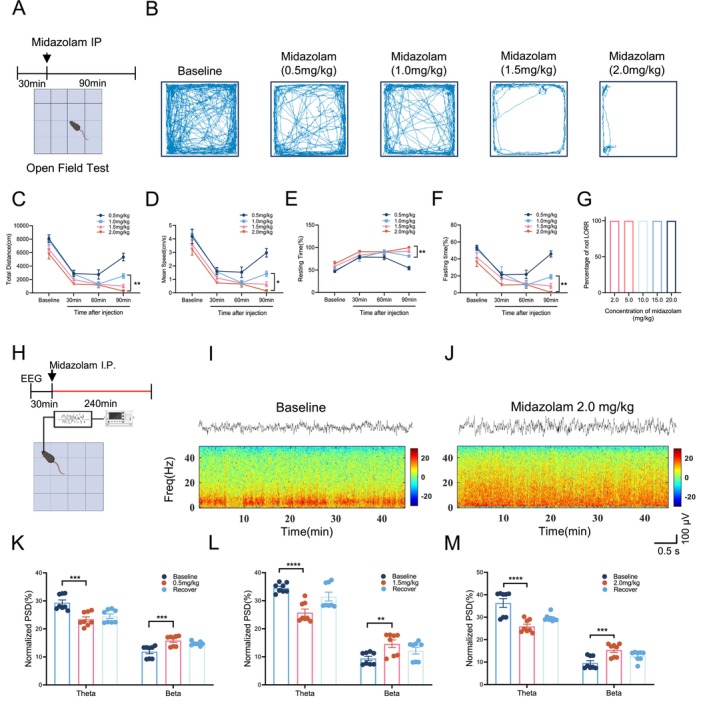
Midazolam produces dose‐and time‐dependent sedation In vivo. (A) Schematic of the open field test. (B) Representative traces of mice before and after intraperitoneal injection of midazolam (left to right: 0.5, 1.0, 1.5 and 2.0 mg·kg^−1^). Statistical results of total distance traveled (C), mean speed (D), percentage of resting time (E), and percentage of fasting time (F) (*n* = 6 mice). (G) Statistical results of the righting reflex test (*n* = 6 mice). (H) Schematic of EEG recording. (*I* and *J*) power spectrogram of baseline and midazolam (2.0 mg·kg^−1^). (L to M) power spectral density of theta (4 to 8 Hz) and beta (15 to 25 Hz) frequency band (left to right: Dose of midazolam: 0.5, 1.5 and 2.0 mg·kg^−1^) (*n* = 8 mice). *p < 0.05, **p < 0.01, ***p < 0.001, and ****p < 0.0001 by a two‐way ANOVA with Bonferroni correction (*C* to *F*) or a one‐way ANOVA with Bonferroni correction (*K* to *M*).

We validated the pharmacological effects of midazolam in mice. Representative locomotion trajectories of mice before and after injection of different doses of midazolam are showed in Figure 1A,B. The total travel distance (by two‐way ANOVA: *p* = 0.0036 for interaction between groups × time, F[9,60] = 3.151; Figure 1C), mean speed (by two‐way ANOVA: *p* = 0.0131 for interaction between groups × time, F[9,80] = 2.531; Figure 1D) and percentage of fasting time (by two‐way ANOVA: *p* = 0.0010 for interaction between groups × time, F[9,60] = 3.703; Figure 1E) were all decreased, while the percentage of resting time (by two‐way ANOVA: *p* = 0.0010 for interaction between groups × time, F[9,60] = 3.703; Figure 1F) was increased. The dose ‐dependent of midazolam were most evident during the recovery phase. Ninety minutes post‐administration, locomotor capacity recovered in mice treated with low doses, but not in those receiving 2.0 mg·kg^−1^ (1.0 vs. 2.0 mg·kg^−1^: distance: *p* = 0.009; mean speed: *p* = 0.020; fasting time: *p* = 0.005; resting time: *p* = 0.005). The righting reflex test indicated that the doses used in this study produced sedation but did not induce anesthesia in mice (Figure 1G). We also monitored typical EEG changes induced by midazolam (Figure 1H–J), which manifested as a decrease in theta (4–8 Hz) power spectral density (0.5 mg·kg^−1^: *p* = 0.0002; 1.5 mg·kg^−1^: *p* < 0.0001; 2.0 mg·kg^−1^: *p* < 0.0001) and an increase in beta (15–25 Hz) power spectral density (0.5 mg·kg^−1^: *p* = 0.0003; 1.5 mg·kg^−1^: *p* = 0.0082; 2.0 mg·kg^−1^: *p =* 0.0006) (Figure 1K–M).

### 3.2 Midazolam Suppresses Pyramidal Neuron Activity in the AC in a Dose ‐Dependent Manner

**FIGURE 2:**
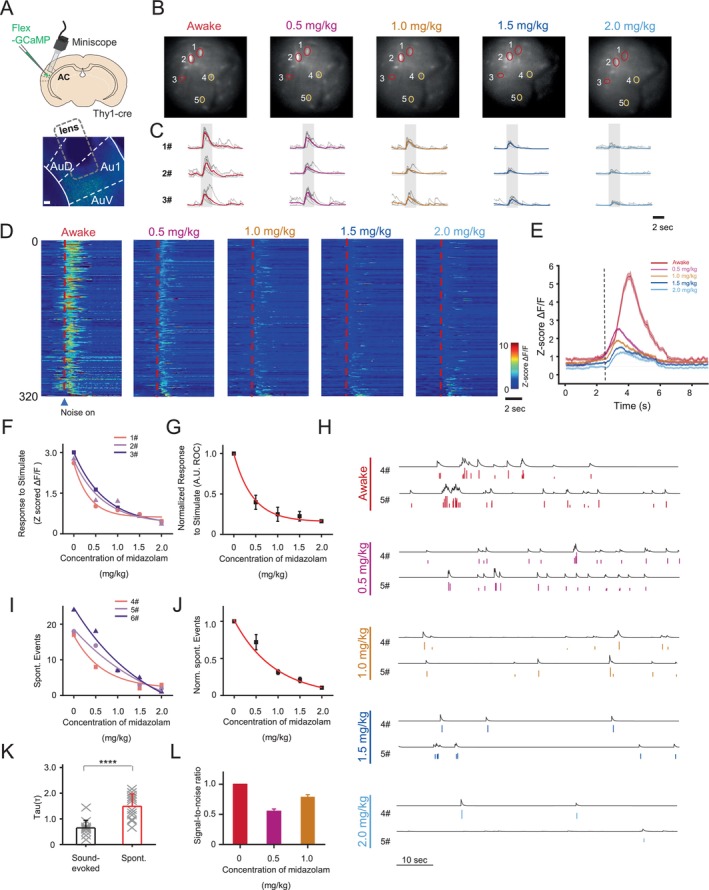
Midazolam suppresses pyramidal neuron activity in the AC in a dose‐dependent manner. (A) Schematic of microendoscopic calcium imaging (top) in AC and representative confocal image showing GCaMP8s expression in deep layers of AC (bottom). (B) Representative field of view (FOV) of GCaMP8s‐expressing neurons under different midazolam doses (0, 0.5, 1.0, 1.5, 2.0 mg·kg^−1^). (C) Calcium signal AC from the FOV shown in (*B*) (red), aligned to the noise onset (gray: Noise, 70 dB, 2 s). (D) Ca^2+^ response heatmap. Grouped by midazolam dose, from left to right are awake, 0.5, 1.0, 1.5, 2.0 mg·kg^−1^. Each row represents the calcium signal response of a neuron, with a red dotted line indicating the start of the sound (*n* = 320 neurons from 10 mice). (E) The mean response curve of the peak value of calcium transient induced by AC pyramidal neuron noise at different doses of midazolam. (F) Averaged calcium signal peaks from three representative trials at different midazolam doses. (G) Normalized response curve of noise‐evoked calcium transient peaks in AC pyramidal neurons across midazolam doses. (H) Spontaneous calcium signal from representative neurons in (B) (yellow) under different midazolam doses. (*I* and *J*) Analysis of spontaneous events corresponding to (F) and (G). (K) Mean time constant (*τ*) of evoked versus spontaneous responses (Mann–Whitney *U*‐test, *p* < 0.001; *n* = 10 neurons). (L) Signal‐to‐noise ratio (SNR) across midazolam doses. (***p < 0.001).

Accumulating evidence suggests that anesthetics suppress activity in the AC, prompting us to investigate how midazolam affects pyramidal neurons in this region. We expressed GCaMP8s in pyramidal neurons of the AC in Thy1‐Cre mice and implanted a GRIN lens at a 15°–20° tilt to minimize damage to cortical columns (Figure 2A). Mice were head‐fixed on the platform and received intraperitoneal injections of different doses of midazolam during recordings. As shown in three example neurons, the sound‐evoked Ca^2+^ signals progressively decreased with increasing doses of midazolam (Figure 2B,C). We quantified neuronal activity by calculating the change in the fluorescent signal (Δ*F/F*) before and after the acoustic stimulation and generated a heatmap to illustrate changes in neuronal responses under different drug doses (Figure 2D). As the dose of midazolam increased, the response of pyramidal neurons to sound stimulation gradually weakened, and their reliability decreased. This trend was further validated by calculating the response amplitude (Figure 2E). Exponential curve fitting of the peak amplitude of Z‐scored Δ*F/F* values against the dose of midazolam further revealed that midazolam inhibits the auditory response in a dose‐dependent manner (Figure 2F,G), The fitted τ values were used as an index of the apparent sensitivity of neuronal responses to increasing midazolam doses. We next analyzed spontaneous activity using a fast online active set method reported in a previous study [32], which was used to extract neural activity from raw fluorescence calcium imaging data (Figure 2H). The results showed that spontaneous activity also decreased progressively with increasing doses of midazolam (Figure 2I,J). Comparison of the *τ* values (representing the decay rate of sound‐evoked calcium signals with increasing drug dose) of the fitted curves revealed that midazolam had a stronger effect on evoked activity than spontaneous activity in pyramidal neurons (Evoked vs. Spontaneous: 0.65 ± 0.30 vs. 1.49 ± 0.48, *p* < 0.001) (Figure 2K). Furthermore, the signal‐to‐noise ratio decreased and reached its lowest level at 0.5 mg·kg^−1^ (Figure 2L). Together, these findings demonstrate that midazolam dose‐dependently reduces both sound‐evoked and spontaneous activity of pyramidal neurons in the AC.

### 3.3 Midazolam Also Reduces Pyramidal Neuronal Activity in the ACC

**FIGURE 3:**
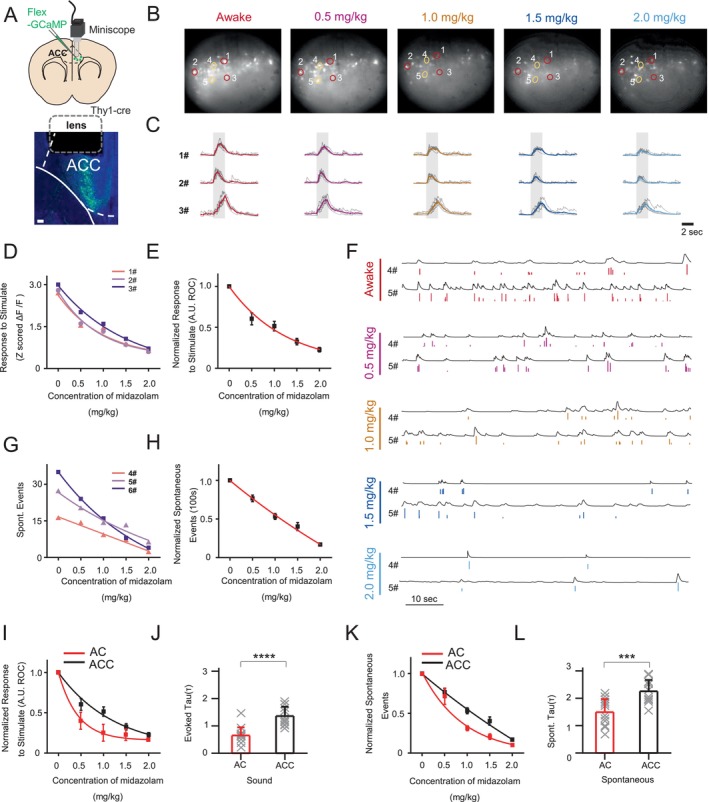
Midazolam also reduces pyramidal neuronal activity in the ACC. (A) Schematic of microendoscopic calcium imaging (top) in ACC and representative confocal image showing GCaMP8s expression (bottom). (B) Representative field of view (FOV) of GCaMP8s‐expressing neurons under different midazolam doses in ACC. (C) Calcium signal traces from the FOV in (*B*) (red), aligned to the noise onset (gray: Noise, 70 dB, 2 s). (D) Averaged calcium signal peaks from three representative trials. (E) Normalized response curve of noise‐evoked calcium transient peaks. (F) Spontaneous calcium signal traces from representative neurons in (*B*) (yellow). (G and H) Analysis of spontaneous events corresponding to (F). (I) Normalized noise‐evoked response peaks in AC (red) versus ACC (black) pyramidal neurons across midazolam doses. (J) Mean time constant (*τ*) of evoked responses (Mann–Whitney *U*‐test; *n* = 10 neurons). (K) Normalized spontaneous response peaks in AC (red) versus ACC (black) pyramidal neurons across midazolam doses. (L) Mean time constant (*τ*) of spontaneous responses (two‐tailed unpaired Student's *t*‐test; *n* = 12 neurons) (***p < 0.001).

To examine whether midazolam also affects excitatory neuronal activity in the ACC, we used microendoscopic Ca^2+^ imaging in this region under different doses of midazolam (Figure 3A). As in the AC, neuronal population activity evoked by noise in the ACC also progressively decreased with increasing doses of midazolam (Figure 3B–E). Similarly, spontaneous calcium transients also exhibited progressive suppression with increasing doses of midazolam (Figure 3F–H). Quantitative comparison showed that the magnitude of suppression for both evoked activity (Figure 3I,J) and spontaneous activity (Figure 3K,L) was greater in the AC than in the ACC (evoked activity: 0.65 ± 0.30 vs. 1.36 ± 0.34, *p* < 0.001; spontaneous activity: 1.49 ± 0.48 vs. 2.25 ± 0.42, *p* < 0.001). Overall, within the tested dose range, midazolam‐induced suppression exhibited significant regional differences, with more pronounced suppression in the AC than in the ACC.

### 3.4 PV ^+^ Interneuron Activity Is Reduced In Vivo in Both the AC and ACC, but to a Lesser Extent Than Pyramidal Neurons Activity

**FIGURE 4:**
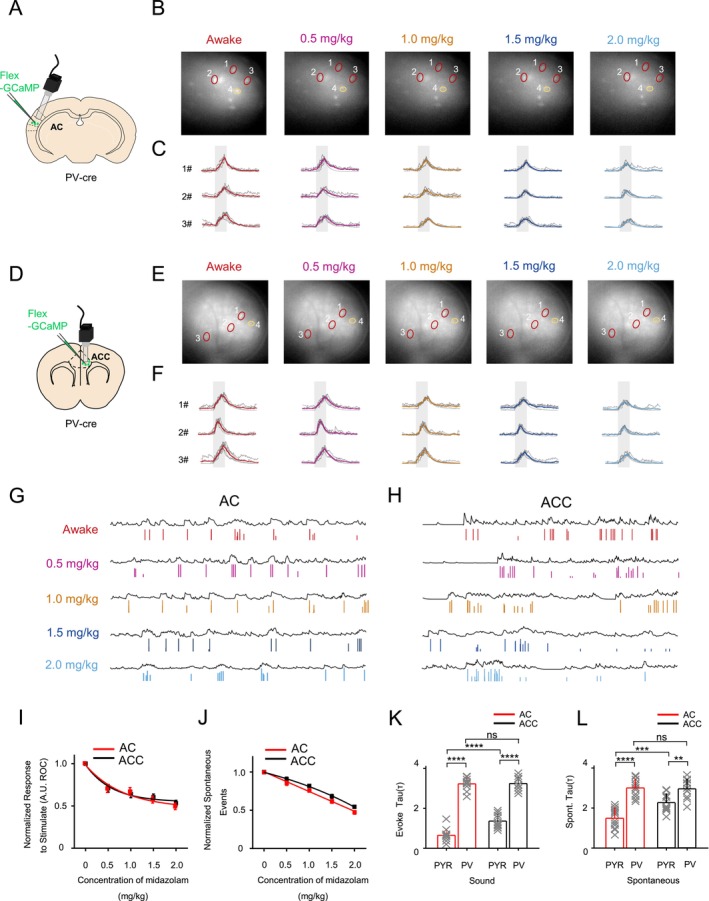
PV^+^ interneuron activity is reduced In vivo in both the AC and ACC, but to a lesser extent than pyramidal neuron activity. (A) Schematic of microendoscopic calcium imaging in AC. (B) Representative field of view (FOV) of GCaMP8s‐expressing neurons under different doses of midazolam. (C) Calcium signal traces from the same FOV shown in (B) (red), aligned to the noise onset. (D) Schematic of microendoscopic calcium imaging in ACC. (E) same as (B). (F) Calcium signal traces from the same FOV shown in (E) (red), aligned to the noise onset. (G) Single‐trial spontaneous calcium transients from PV^+^ neurons in AC. (H) Single‐trial spontaneous calcium transients from PV^+^ neurons in ACC. (I) Normalized evoked response of AC PV^+^ neurons(red) versus ACC PV^+^ neurons (black). (J) Normalized spontaneous events of AC PV^+^ neurons(red) versus ACC PV^+^ neurons (black). (K) Mean time constant (τ) of evoked response of pyramidal neurons versus PV^+^ neurons in AC and ACC. (L) Mean time constant (τ) of spontaneous events of pyramidal neurons versus PV^+^ neurons in AC and ACC. (Mann–Whitney *U*‐test and two‐tailed unpaired *t*‐test; *n* = 10 neurons). (*p < 0.05, **p < 0.01, ***p < 0.001).

Given the observed effects on pyramidal neurons, we also examined the impact of midazolam on inhibitory interneurons. We imaged PV^+^ neurons in the AC (Figure 4A) and the ACC (Figure 4D). Both sound‐evoked Ca^2+^ signals and spontaneous calcium events in PV^+^ neurons in the AC (Figure 4B,C,G) and the ACC (Figure 4E,F,H) were attenuated with increasing dose of midazolam. However, unlike the regional differences observed in excitatory neurons, the attenuation curves showed no significant difference between AC and ACC PV^+^ neurons (Figure 4I,J). Notably, the reduction in both signal types was greater in pyramidal neurons than in PV^+^ interneurons (Figure 4K,L). In the AC, the reduction in evoked activity was greater in pyramidal neurons (0.65 ± 0.30) than in PV^+^ neurons (3.23 ± 0.28; *p* < 0.001), and a similar pattern was observed for spontaneous activity (1.49 ± 0.48 vs. 3.00 ± 0.41; *p* < 0.001). Similarly, in the ACC, reductions in evoked activity (1.36 ± 0.34 vs. 3.24 ± 0.30; *p* < 0.001) and spontaneous activity (2.25 ± 0.42 vs. 2.94 ± 0.44; *p* = 0.001) were both greater in pyramidal neurons than in PV^+^ neurons.

### 3.5 Midazolam Reduces the Intrinsic Excitatory of Pyramidal Neurons in Both the AC and ACC in Acute Brain Slices

**FIGURE 5:**
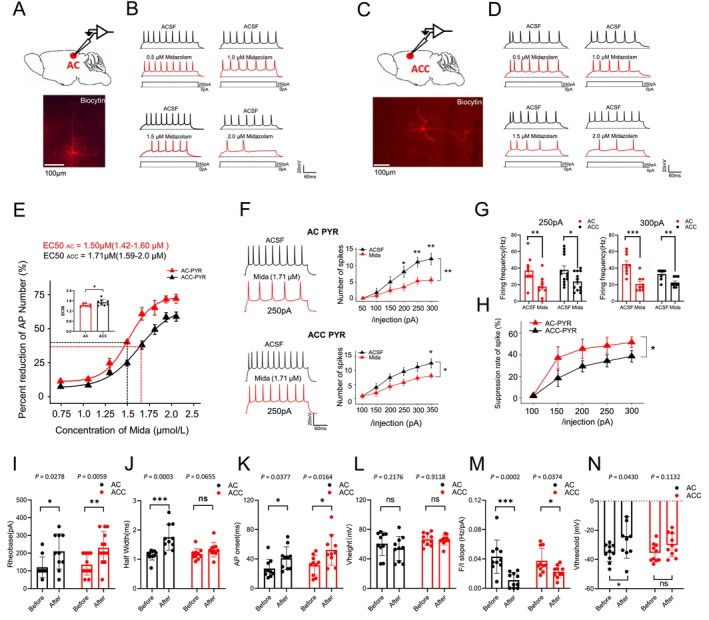
Midazolam reduces the intrinsic excitatory activity of pyramidal neurons in both the AC and ACC in acute brain slices. (A) Upper: Schematic of whole‐cell patch‐clamp recording in AC pyramidal neurons. Lower: Images showing pyramidal neurons in AC labeled with biocytin (scale bar = 100 μm). (B) Representative action potential (AP) traces evoked by 250 pA current injection in AC pyramidal neurons. Pre‐midazolam (black) vs. post‐midazolam (red). Midazolam concentrations (0.5, 1, 1.5, 2 μM). Scale bar: 60 ms, 20 mV. (C) same as (*A*) in ACC. (D) same as (*B*) in ACC. (E) Concentration‐response curves for midazolam‐induced AP suppression in AC (red) and ACC (black) pyramidal neurons (*n* = 8 neurons/3 mice). Insert: EC50 comparison (*n* = 8 neurons/3 mice). (F) Upper: AP traces in AC pyramidal neurons pre‐ (black) and post‐(red) midazolam (1.71 μM) at 250 pA. Lower: Corresponding traces in ACC pyramidal neurons. (AC: *N* = 10 neurons/3 mice; ACC: *N* = 13 neurons/4 mice). (G) Firing frequency of AC and ACC caused by Midazolam which inject current at 250 and 300pA. (H) Midazolam‐induced firing rate suppression in AC and ACC pyramidal neurons (*n* = 10 neurons/3 mice). (*I* to *M*) Midazolam's effects on electrophysiological parameters: Rheobase (I), Half width (J), AP onset (K), Vheight (L), F/I slope (*M*) and AP threshold (*N*) (*n* = 10 neurons/3 mice). *p < 0.05, **p < 0.01, ***p < 0.001 by a two‐way repeated‐measures ANOVA with Bonferroni correction (*F*and *H*) or a two‐tailed unpaired *t*‐test (G, J, Kand N) or a Mann–Whitney *U*‐test (I, Land M).

To examine the direct effects of midazolam on intrinsic excitability, we performed whole‐cell patch‐clamp recordings in acute brain slices from the AC (Figure 5A) and ACC (Figure 5C). Midazolam (0.5–2.0 μM) decreased the firing frequency of pyramidal neurons in a concentration‐dependent manner in both regions (Figure 5B,D). By comparing the concentration‐response curves of neurons from the two brain regions, we found that the EC50 value in the AC (1.50 μM, 95% CI, 1.42 to 1.60 μM) was significantly lower than that in the ACC (1.71 μM, 95% CI, 1.59 to 2.0 μM) (AC vs. ACC: 1.54 ± 0.09 vs. 1.70 ± 0.17, *p* = 0.041) (Figure 5E). We further examined the effects of midazolam on neuronal excitability using incremental current injections. In AC pyramidal neurons, midazolam significantly reduced action potential frequency at 50‐ to 300‐ pA steps (*p* = 0.005, F [1, 18] = 10.34), showing maximal suppression at 300 pA (*p* = 0.001). ACC pyramidal neurons also showed a significant reduction in firing rate (*p* = 0.029, F [1, 24] = 5.43) (Figure 5F). Notably, AC neurons showed stronger drug‐induced firing suppression than ACC neurons (*p* = 0.028, F [1, 21] = 5.57) (Figure 5G,H). In addition, key electrophysiological properties showed minor or no regional differences (Figure 5I–N): Rheobase, AC (120 ± 55.86 to 210 ± 94.34 pA; *p* = 0.028), ACC (134.62 ± 56.79 to 230.77 ± 86.69 pA; *p* < 0.001); Half‐width, AC (1.15 ± 0.17 to 1.75 ± 0.42 ms; *p* < 0.001), ACC (1.17 ± 0.18 to 1.33 ± 0.24 ms; *p* = 0.07); AP onset, AC (26.83 ± 11.74 to 40.99 ± 14.86 ms; *p* = 0.038), ACC (31.58 ± 12.73 to 52.38 ± 19.86 ms; *p* = 0.016); Vheight, AC (60.63 ± 15.43 to 54.12 ± 15.63 mV; *p* = 0.218), ACC (66.58 ± 7.81 to 65.55 ± 6.43 mV; *p* = 0.91); F/I slope, AC (0.04 ± 0.01 to 0.01 ± 0.003 Hz/pA; *p* < 0.001), ACC (0.04 ± 0.01 to 0.02 ± 0.003 Hz/pA; *p* = 0.037); Threshold, AC (−35.54 ± 5.62 to −24.91 ± 13.52 mV; *p* = 0.043), ACC (−35.46 ± 5.99 to −30.15 ± 7.45 mV; *p* = 0.11). Collectively, midazolam showed differential effects on pyramidal neurons across regions in vitro, consistent with the in vivo observations.

We next investigated the effect of midazolam on the intrinsic excitability of PV^+^ interneurons in the AC and ACC in vitro (Figure S1A–D). In contrast to pyramidal neurons, midazolam did not significantly alter the intrinsic excitability of PV+ interneurons in either AC or ACC slices (AC: p = 0.225, F[1, 24] = 1.55; ACC: p = 0.447, F[1, 24] = 0.60) (Figure S1B,D). Consistent with this, midazolam did not significantly reduce PV+ interneuron firing in either region (AC: 118.79 ± 8.30 vs. 103.64 ± 11.25, p = 0.291; ACC: 115.90 ± 4.75 vs. 107.44 ± 5.33, p = 0.247), and no significant inter‐regional difference was detected (p = 0.423, F[1, 21] = 0.66) (Figure S1E,F). This limited effect persisted at higher concentrations (Figure S1G). Most other electrophysiological properties of PV+ interneurons were unchanged after midazolam application in both regions (Figure S1H–M): Rheobase, AC (75 ± 43.30 to 95.83 ± 59.37, p = 0.27), ACC (95.45 ± 33.40 to 100 ± 30.15, p = 0.85); AP half‐width, AC (0.72 ± 0.12 vs. 0.77 ± 0.38, p = 0.374), ACC (0.78 ± 0.17 to 0.78 ± 0.09, *p* = 0.976); AP onset, AC (15.46 ± 3.36 to 15.84 ± 3.56, *p* = 0.819), ACC (21.21 ± 8.90 to 22.53 ± 6.55, *p* = 0.724); AP amplitude, AC (51.81 ± 11.09 to 50.94 ± 14.19, *p* = 0.724), ACC (40.79 ± 8.97 to 42.41 ± 9.53, *p* = 0.755); F/I slope, AC (0.15 ± 0.03 to 0.13 ± 0.03, *p* = 0.191), ACC (0.08 ± 0.02 to 0.08 ± 0.02, *p* = 0.792), and AP threshold, AC (−42.79 ± 3.24 to −40.91 ± 8.32, *p* = 0.97), ACC (−37.46 ± 3.43 to −41.23 ± 3.32, *p* = 0.03). Thus, under these slice recording conditions, midazolam had limited direct effects on the intrinsic excitability of PV+ interneurons in either region.

### 3.6 Differential GABRA1 Expression May Be Associated With Midazolam's Brain Region‐Specific Effects

**FIGURE 6:**
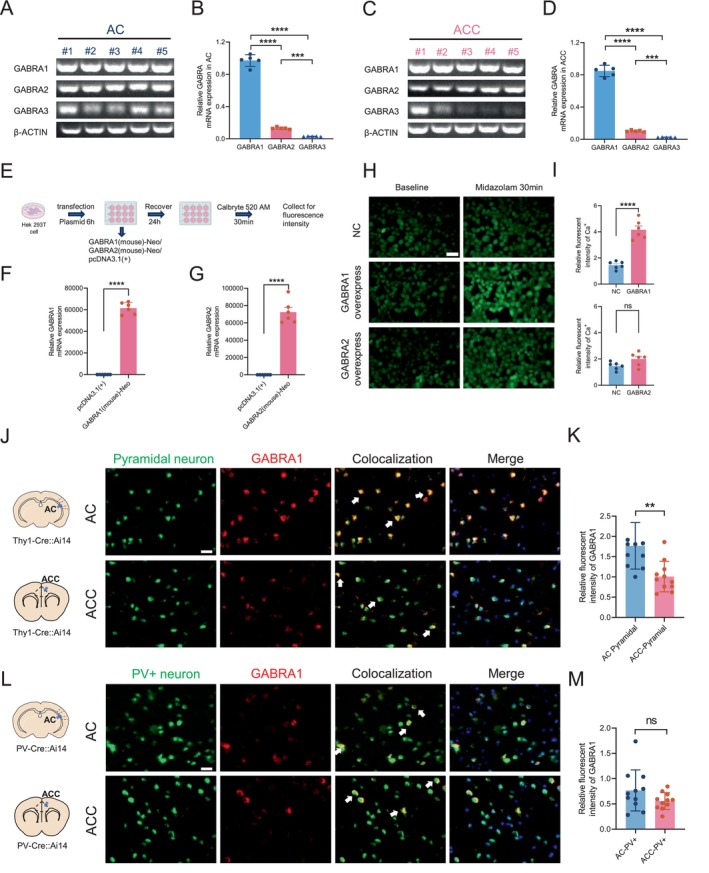
Differential GABRA1 expression may be associated with midazolam's brain region‐specific effects. (A and C) Schematic of RT‐qPCR analysis comparing mRNA levels of GABAARs (GABRA1/2/3) in AC and ACC. (B and D) Quantitative analysis of cortical regions reveals differential transcriptional levels among GABAARs in AC and ACC (*n* = 5 biological replicates/15 mice total; 3 mice per replicate). (E) Experimental workflow for plasmid transfection and calcium imaging in HEK293T cells. (F and G) Statistical validation of successful GABRA1 (F) and GABRA2 (G) overexpression. (H) Schematic of midazolam‐induced calcium flux measurement in transfected cells. Scale bar = 200 μm. (I) Midazolam induced intracellular calcium changed in GABRA1 and GABRA2 overexpressing cells. (J) Immunohistochemical staining of GABRA1 in AC and ACC pyramidal neurons. (*K*) Quantitative analysis of GABRA1 in AC and ACC pyramidal neurons. (images = 9 from 3 mice). (*L*) Immunohistochemical staining of GABRA1 in AC and ACC PV^+^ neurons. (M) Quantitative analysis of GABRA1 in AC and ACC PV^+^ neurons. (images = 9 from 3 mice). Scale bar = 50 μm.* *p* < 0.05, **p < 0.01, ***p < 0.001 by a Owo‐way ANOVA with Bonferroni posttest (*B* and *C*) or a two‐tailed unpaired *t*‐test (F, G,I, K and M).

Midazolam exerts its effects through GABAARs, although the specific receptor subtypes responsible remain a topic of debate [1]. To address this, we first examined the expression levels of three major GABAAR subtypes (GABRA1, GABRA2, and GABRA3) in the AC and ACC tissues. RT‐qPCR analysis revealed that GABRA1 expression was significantly higher than that of GABRA2 or GABRA3 in both regions (AC: GABRA1 vs. GABRA2, 0.97 ± 0.06 vs. 0.13 ± 0.01, *p* < 0.0001; GABRA1 vs. GABRA3, 0.97 ± 0.06 vs. 0.03 ± 0.003, *p* < 0.0001; ACC: GABRA1 vs. GABRA2, 0.85 ± 0.06 vs. 0.10 ± 0.01, *p* < 0.0001; GABRA1 vs. GABRA3, 0.85 ± 0.06 vs. 0.03 ± 0.001, *p* < 0.0001) (Figure 6A–D). To assess the relationship between GABRA1 abundance and midazolam responsiveness, we overexpressed GABRA1 or GABRA2 in HEK293T cells using plasmid constructs (Figure 6E). RT‐qPCR confirmed successful overexpression (GABRA1: vector vs. GABRA1‐neo, 0.59 ± 0.19 vs. 61449.19 ± 4931.55, *p* < 0.0001; GABRA2: vector vs. GABRA2‐neo, 0.43 ± 0.27 vs. 72335.73 ± 12251.93784, *p* < 0.0001) (Figure 6F,G). Calcium imaging using a fluorescent probe revealed that midazolam evoked a significantly stronger intracellular Ca^2+^ response in GABRA1‐overexpressing cells than in control cells (fluorescence intensity: 1.45 ± 0.27 vs. 4.16 ± 0.67, *p* < 0.0001), whereas the increase in GABRA2‐overexpressing cells did not reach significance (1.47 ± 0.27 vs. 1.99 ± 0.47, *p* = 0.057) (Figure 6H,I). To examine whether the differential responses to midazolam in the AC and ACC were associated with differences in membrane‐localized GABRA1, we performed immunofluorescence staining in these two regions from Thy1‐Cre; Ai14 mice (for pyramidal neurons) and PV‐Cre; Ai14 mice (for PV^+^ interneurons). Notably, pyramidal neurons in the AC exhibited significantly greater GABRA1 expression than those in the ACC (1.77 ± 0.55 vs. 1.01 ± 0.36, *p* = 0.002) (Figure 6J,K). In contrast, GABRA1 expression in PV^+^ neurons did not significantly differ between the AC and ACC (0.77 ± 0.39 vs. 0.56 ± 0.16, *p* = 0.129) (Figure 6L,M). These findings suggest that regional differences in GABRA1 expression may contribute to differential sensitivity to midazolam between the AC and ACC.

## 4 Discussion

In this study, we examined region‐ and cell‐type‐specific cortical responses to midazolam in the neocortex, focusing on the AC and ACC. Combined miniscope Ca^2+^ imaging and acute slice recordings revealed that midazolam exerted a stronger suppressive effect on excitatory pyramidal neurons in the AC than in the ACC, while PV^+^ interneurons showed smaller activity changes in vivo and limited changes in intrinsic excitability in vitro in either region. Molecular profiling further indicated that this pattern was associated with regional differences in GABRA1 expression.

Cortical activity relies on the dynamic excitatory‐inhibitory (E/I) balance mediated by glutamatergic and GABAergic neurotransmission [33]. Multiple anesthetics and sedatives alter E/I balance and are associated with sedation, unconsciousness, or motor suppression [34, 35]. Agents such as isoflurane, propofol, and diazepam enhance GABAAR‐mediated inhibition, thereby reducing neuronal excitability and promoting cortical network states characterized by low‐frequency synchrony and reduced overall activity [36]. Conversely, ketamine antagonizes NMDA receptors, reducing excitatory drive and the E/I ratio [37]. In this study, we observed cell‐type‐specific effects of midazolam on cortical neurons. Pyramidal neurons in the AC and ACC showed concentration‐dependent reductions in intrinsic excitability in response to midazolam. In contrast, in vitro patch‐clamp recordings showed that PV^+^ interneurons exhibited limited changes, with firing properties remaining relatively stable even at high drug concentrations. In vivo miniscope Ca^2+^ imaging revealed moderate reductions in spontaneous and sound‐evoked activity of PV^+^ interneurons, although the magnitude of these reductions remained significantly smaller than that observed in excitatory neurons. One possible explanation is that midazolam may modulate inhibitory neurons in upstream nuclei that influence cortical PV^+^ interneurons, thereby indirectly increasing inhibitory input onto PV^+^ cells. In this case, the reduction in PV^+^ activity observed in vivo would reflect decreased excitatory drive and broader network‐level influences rather than a strong direct effect of midazolam on PV^+^ interneurons. These findings are consistent with a shift in the local E/I balance, whereas inhibitory activity appears to be less strongly affected. Such a shift may contribute to reduced cortical activity under midazolam, although its relationship to the broader features of sedative states remains to be determined [38].

General anesthetics are known to exert regionally heterogeneous effects across the cortex [39]. Clinical EEG and functional MRI studies have shown that during anesthesia‐induced unconsciousness, higher‐order association cortices (such as the prefrontal cortex) often exhibit disrupted connectivity, while activity in primary sensory cortices may be preserved until deeper anesthetic states are reached [5, 40]. Animal and clinical studies further suggest regional differences in cortical sensitivity. Sensory cortices, such as the visual and auditory areas, exhibit significant attenuation of stimulus‐evoked responses under agents such as isoflurane and propofol [41, 42]. However, whether such region‐specific effects extend to sedatives remains poorly understood. In our study, midazolam reduced activity in pyramidal neurons of the AC more strongly than in those of the ACC, for both sound‐evoked and spontaneous responses. This finding suggests region‐specific sensitivity to sedatives at the cellular level. One possible interpretation is that this regional difference may be relevant to how sedative agents affect distinct cortical subsystems. However, the present study did not directly assess sensory perception or higher‐order cognitive function, and therefore such functional implications remain speculative. Further studies will be required to determine whether these regional differences contribute to distinct features of sedative states.

To investigate the cell‐type‐ and region‐dependent effects of midazolam, we focused on its pharmacological target, GABAARs. Benzodiazepines bind to α‐γ subunit interfaces, and α‐subunit heterogeneity contributes to drug selectivity and pharmacodynamics [43, 44]. We identified differential expression of three α subunits in both the AC and ACC, with GABRA1 levels substantially higher than those of GABRA2 and GABRA3. Overexpression experiments supported an association between GABRA1 abundance and midazolam responsiveness, consistent with in vivo findings from Puig‐Bosch et al. [45]. GABAAR expression levels and spatial organization may contribute to cell‐type differences in midazolam responsiveness. Differential GABRA1 expression in AC and ACC neurons of transgenic mice may be associated with the region‐dependent effects of midazolam. In our dataset, glutamatergic neurons exhibited higher GABRA1 expression than GABAergic neurons, which is consistent with the possibility that differential GABRA1 distribution may contribute to neuron‐type‐dependent responses to midazolam. Previous studies have shown that GABAARs composed of α1β2δ subunits mediate tonic inhibition in PV^+^ interneurons and are selectively expressed in these neurons [46]. These observations are consistent with the idea that specific GABAAR subtypes and their spatial distribution may contribute to differential cellular responses to anesthetic drugs.

Study limitations include the potential influence of presynaptic modulation on neuronal activity during sedation, which requires further investigation to elucidate the underlying mechanisms. In acute brain slices, the loss of much of the intact recurrent and long‐range excitatory input leads to markedly reduced network activity, consequently diminishing ongoing GABAergic tone. Therefore, the strength of tonic inhibition is likely substantially underestimated under slice conditions. The predominantly “quiescent” state of PV^+^ neurons and other neuronal populations in slices makes it challenging to comprehensively evaluate the contribution of tonic inhibition under these. This limitation may partly explain why midazolam continued to suppress pyramidal neurons under slice conditions, whereas its direct effects on PV^+^ interneuron intrinsic excitability appeared limited under the same conditions. The effects of midazolam may depend in part on the distribution of its target receptors. Thus, differences in tonic inhibition alone are unlikely to fully account for the cell‐type‐specific effects observed here. The region‐ and cell‐type‐specific effects of midazolam may depend in part on differences in specific GABAAR subtypes, necessitating further functional studies beyond GABRA1 expression density.

In summary, our study refines the understanding of the cortical effects of midazolam. Previous studies, including those on midazolam, have often described broad cortical suppression. In contrast, we found that midazolam produced region‐ and cell‐type‐specific effects, with stronger effects in the AC than in the ACC. This finding suggests that the cortical effects of midazolam are not uniform across regions and cell types. These effects were associated with differential GABRA1 expressions, with pyramidal neurons in the AC showing greater sensitivity to midazolam and higher GABRA1 levels. Our findings provide a more specific framework for understanding how midazolam differentially affects cortical neuronal populations.

## Author Contributions

Each author played a crucial role in the successful completion of this study. Hong‐Fei Zhang, Feixue Liang, Junlei Chang, and Fengxian Li designed the study and drafted the manuscript. Yao Sun, Dijia Wang, Kaibin Wu, Mengyu Yin, and Peiwen Tang collected and analyzed the data. All authors reviewed the manuscript. All authors reviewed and agreed to submit the manuscript.

## Funding

This work was supported by the National Natural Science Foundation of China (82471294) (32271061) (32471062). (Science and Technology Innovation 2030 MajorProjects, China) (2021ZD0202600). National Natural Science Foundation of China‐Guangdong Joint Fund (2024A1515012256). Guangdong‐Hong Kong Joint Laboratory for Psychiatric Disorders (2023B1212120004). Guangzhou Science and Technology Plan Project (202206060004).

## Ethics Statement

Animal experiments were conducted in accordance with the guidelines of the Chinese Association for Laboratory Animal Sciences and were approved by the Animal Care and Use Committee of Southern Medical University (Approval No. L2017207).

## Conflicts of Interest

The authors declare no conflicts of interest.

## Supporting information


**Figure S1:** Midazolam does not significantly alter the intrinsic excitability of PV^+^ interneuron in the AC and ACC in vitro.
(*A*) Left: Schematic of whole‐cell patch‐clamp recording in PV^+^ neurons in the AC. Right: Fluorescently labeled PV^+^ neurons in the AC of a PV‐Cre::Ai14 mouse (scale bar = 500 μm). (*B*) Left: Representative action potential (AP) traces in AC PV^+^ neurons pre‐ (black) and post‐midazolam (1.71 μM, red) at 250 pA. Right: Spike counts across 50‐ to 300‐ pA current injections (*n* = 10 neurons/3 mice). (*C*) Same as (*A*) in the ACC. (*D*) Same as (*B*) in the ACC (*n* = 13 neurons/3 mice). (*E*) Firing frequencies of PV^+^ neurons in the AC and ACC after midazolam application (1.71 μM) at a 250‐pA current injection. (*F*) Midazolam‐induced firing rate modulation in AC and ACC PV^+^ neurons (*n* = 10 neurons/3 mice). (*G*) Firing frequencies of PV+ neurons in the AC and ACC after midazolam application (2.50 μM) at 250‐ and 300‐pA current injections. (*H* to *M*) Midazolam effects on electrophysiological parameters: Rheobase (*H*), AP half‐width (*I*), AP onset (*J*), AP height (*K*), F/I slope (*L*) and AP threshold (*M*) (*n* = 10 neurons from 3 mice). * *p* < 0.05, ** *p* < 0.01, *** *p* < 0.001 by a two‐way repeated‐measures ANOVA with Bonferroni correction (*B*, *D* and *F*) or a Mann–Whitney *U*‐test (*G*, *H*, *K* and *L*) or a two‐tailed unpaired *t*‐test (*J* and *K*). A two‐way repeated‐measures ANOVA with Bonferroni correction (*F*, *G* and *E*) or a Mann–Whitney *U*‐test (*F*, *I* and *K*) or a two‐tailed unpaired *t*‐test (*E*, *G*, *I* and *J*).

## Data Availability

The data that support the findings of this study are available from the corresponding author upon reasonable request.
