# Supplementary material for: Midazolam Exerts Sedative Effects by Differentially Modulating Cortical Pyramidal Neurons and PV Interneurons
Source: CNS Neurosci Ther. 2026 Jul 24;32(7):e71049. doi: 10.1002/cns.71049 (PMC13398157; doi:10.1002/cns.71049)
Supplement: Supplementary file 1 — Figure S1: Midazolam does not significantly alter the intrinsic excitability of PV+ interneuron in the AC and ACC in vitro. (A) Left: Schematic of whole‐cell patch‐clamp recording in PV+ neurons in the AC. Right: Fluorescently labeled PV+ neurons in the AC of a PV‐Cre::Ai14 mouse (scale bar = 500 μm). (B) Left: Representative action potential (AP) traces in AC PV+ neurons pre‐ (black) and post‐midazolam (1.71 μM, red) at 250 pA. Right: Spike counts across 50‐ to 300‐ pA current injections (n = 10 neurons/3 mice). (C) Same as (A) in the ACC. (D) Same as (B) in the ACC (n = 13 neurons/3 mice). (E) Firing frequencies of PV+ neurons in the AC and ACC after midazolam application (1.71 μM) at a 250‐pA current injection. (F) Midazolam‐induced firing rate modulation in AC and ACC PV+ neurons (n = 10 neurons/3 mice). (G) Firing frequencies of PV+ neurons in the AC and ACC after midazolam application (2.50 μM) at 250‐ and 300‐pA current injections. (H to M) Midazolam effects on electrophysiological parameters: Rheobase (H), AP half‐width (I), AP onset (J), AP height (K), F/I slope (L) and AP threshold (M) (n = 10 neurons from 3 mice). * p < 0.05, ** p < 0.01, *** p < 0.001 by a two‐way repeated‐measures ANOVA with Bonferroni correction (B, D and F) or a Mann–Whitney U‐test (G, H, K and L) or a two‐tailed unpaired t‐test (J and K). A two‐way repeated‐measures ANOVA with Bonferroni correction (F, G and E) or a Mann–Whitney U‐test (F, I and K) or a two‐tailed unpaired t‐test (E, G, I and J). [file CNS-32-e71049-s001.docx]

**Supplementary material**

**Supplementary Figure1**


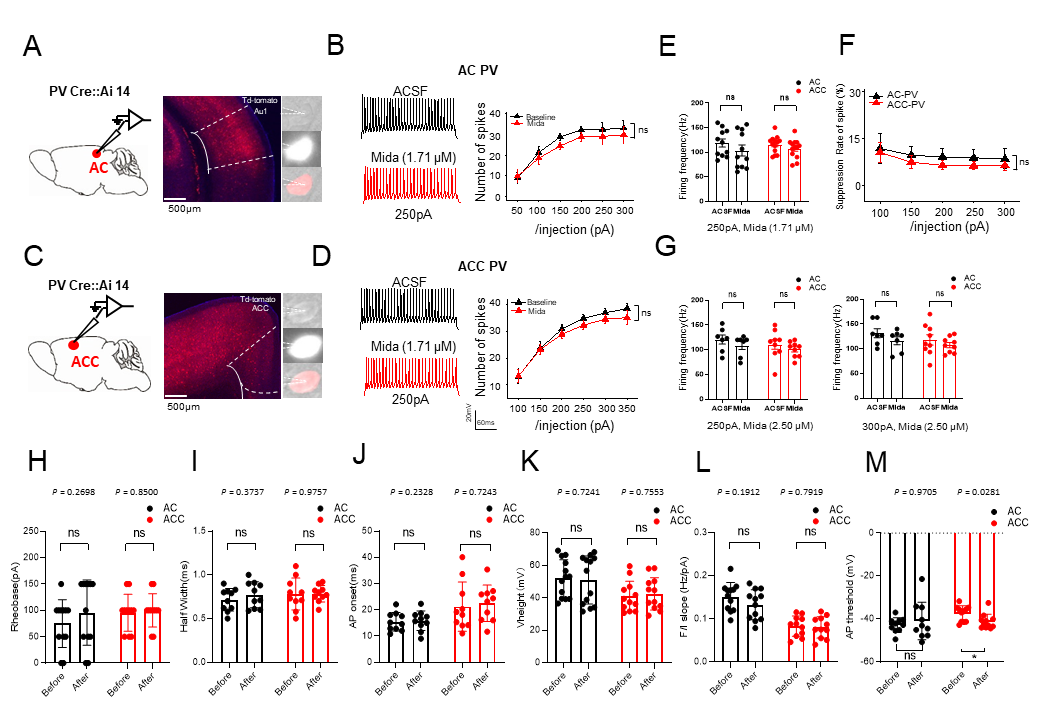


In contrast to pyramidal neurons, midazolam did not significantly affect the intrinsic excitability of PV^+^ interneurons in either AC or ACC slices (AC: *P* = 0.225, F [1, 24] = 1.55; ACC: *P* = 0.447, F[1, 24] = 0.60) (**Supplementary Fig. 1B and D**). There were no differences in firing suppression (AC: 118.79 ± 8.30 *vs.* 103.64 ± 11.25, *P* = 0.291; ACC: 115.90 ± 4.75 *vs.* 107.44 ± 5.33, *P* = 0.247; Inter-region comparison: *P* = 0.423, F[1, 21] = 0.66)(**Supplementary Fig. 1E and F**). This limited effect persisted at higher concentrations **(Supplementary Fig. 1G).** We also found there were no differences in electrophysiological properties (**Supplementary Fig. 1H-M**): Rheobase: AC (75 ± 43.30 to 95.83 ± 59.37, *P* = 0.27), ACC (95.45 ± 33.40 to 100 ± 30.15, *P* = 0.85); AP half-width: AC (0.72 ± 0.12 *vs.* 0.77 ± 0.38, *P* = 0.374), ACC (0.78 ± 0.17 to 0.78 ± 0.09, *P* = 0.976); AP onset: AC (15.46 ± 3.36 to 15.84 ± 3.56, *P* = 0.819), ACC (21.21 ± 8.90 to 22.53 ± 6.55, *P* = 0.724); AP amplitude: AC (51.81 ± 11.09 to 50.94 ± 14.19, *P* = 0.724), ACC (40.79 ± 8.97 to 42.41 ± 9.53, *P* = 0.755); F/I slope: AC (0.15 ± 0.03 to 0.13 ± 0.03, *P* = 0.191), ACC (0.08 ± 0.02 to 0.08 ± 0.02, *P* = 0.792), and AP threshold: AC (-42.79 ± 3.24 to -40.91 ± 8.32, *P* = 0.97), ACC (-37.46 ± 3.43 to -41.23 ± 3.32, *P* = 0.03). Thus, under these slice recording conditions, midazolam had limited direct effects on the intrinsic excitability of PV^+^ interneurons in either region.

**Supplementary Fig1.** **Midazolam does not significantly alter the intrinsic excitability of PV^+^ interneuron in the AC and ACC in vitro.**

(*A*) Left: Schematic of whole-cell patch-clamp recording in PV^+^ neurons in the AC. Right: Fluorescently labeled PV^+^ neurons in the AC of a PV-Cre::Ai14 mouse (scale bar = 500 μm). (*B*) Left: Representative action potential (AP) traces in AC PV^+^ neurons pre- (black) and post-midazolam (1.71 μM, red) at 250 pA. Right: Spike counts across 50- to 300- pA current injections (n = 10 neurons/3 mice). (*C*) Same as (*A*) in the ACC. (*D*) Same as (*B*) in the ACC (n = 13 neurons/3 mice). (*E*) Firing frequencies of PV^+^ neurons in the AC and ACC after midazolam application (1.71 μM) at a 250-pA current injection. (*F*) Midazolam-induced firing rate modulation in AC and ACC PV^+^ neurons (n = 10 neurons/3 mice). (*G*) Firing frequencies of PV+ neurons in the AC and ACC after midazolam application (2.50 μM) at 250- and 300-pA current injections. (*H* to *M*) Midazolam effects on electrophysiological parameters: Rheobase (*H*), AP half-width (*I*), AP onset (*J*), AP height (*K*), F/I slope (*L*) and AP threshold (*M*) (n = 10 neurons from 3 mice). * *P* < 0.05, ** *P* < 0.01, *** *P* < 0.001 by a two-way repeated-measures ANOVA with Bonferroni correction (*B*, *D* and *F*) or a Mann-Whitney U-test (*G*, *H*, *K* and *L*) or a two-tailed unpaired t test (*J* and *K*). A two-way repeated-measures ANOVA with Bonferroni correction (*F*, *G* and *E*) or a Mann-Whitney U-test (*F*, *I* and *K*) or a two-tailed unpaired t test (*E*, *G*, *I* and *J*).
